# Agentic processes in cultural evolution: relevance to Anthropocene sustainability

**DOI:** 10.1098/rstb.2022.0252

**Published:** 2024-01-01

**Authors:** Peter J. Richerson, Robert T. Boyd, Charles Efferson

**Affiliations:** ^1^ Department of Environmental Science and Policy, University of California, Davis, 95616, CA, USA; ^2^ School of Human Evolution and Social Change, Institute of Human Origins, Arizona State University, Tempe, 85281, AZ, USA; ^3^ Faculty of Business and Economics, University of Lausanne, 1015, Lausanne, Switzerland

**Keywords:** cultural evolution, human evolution, cumulative culture, Anthropocene

## Abstract

Humans have evolved culturally and perhaps genetically to be unsustainable. We exhibit a deep and consistent pattern of short-term resource exploitation behaviours and institutions. We distinguish agentic and naturally selective forces in cultural evolution. Agentic forces are quite important compared to the blind forces (random variation and natural selection) in cultural evolution and gene-culture coevolution. We need to use the agentic policy-making processes to evade the impact of blind natural selection. We argue that agentic forces became important during our Pleistocene history and into the Anthropocene present. Human creativity in the form of deliberate innovations and the deliberate selective diffusion of technical and social advances drove this process forward for a long time before planetary limits became a serious issue. We review models with multiple positive feedbacks that roughly fit this observed pattern. Policy changes in the case of large-scale existential threats like climate change are made by political and diplomatic agents grasping and moving levers of institutional power in order to avoid the operation of blind natural selection and agentic forces driven by narrow or short-term goals.

This article is part of the theme issue ‘Evolution and sustainability: gathering the strands for an Anthropocene synthesis’.

## 1. Introduction

The purpose of this contribution is two-fold. First, we want to explain how cumulative cultural evolution (CCE) generated the Anthropocene. For the first time in the history of the Earth, a single species has transformed the biogeochemistry of the planet in the space of a few millennia. Second, on the mitigation side, we want to understand how the basic processes of cultural evolution map onto the applied policy sciences of sustainability. How might the basic science of cultural evolution contribute to the practical problem of ending the destructive tendencies of the Anthropocene? Our basic argument is that CCE has introduced a novel anthropogenic destabilizing element into the dynamics of the global ecosystem and that controlling this instability requires deliberate institutional reform. The critical contribution of cultural evolutionary theory is an understanding of these dynamics and their artificial stabilization.

## 2. An outline of the processes of cultural evolution and gene-culture coevolution

Any evolutionary process has elements of continuity and transformation. Transformative processes are often conceived of as forces that perturb continuity of species and cultures. Some of these forces are non-agentic in the sense that they ‘just happen,’ and others are agentic in the sense that decisions of the organisms involved play an active role. Darwin built the *Origin of Species* around this distinction. Natural selection was the non-agentic outcome of variant organisms living out their lives in a particular environment under which some variants survived better or reproduced more successfully than other variants. Using the agentic process of artificial selection, plant and animal breeders caused dramatic changes in many domesticated lineages in Victorian England. Natural theologians had assumed that all evolution required agency and nominated God as the Supreme Breeder. Darwin argued that natural selection could generate adaptations without agency, but he recognized that in some circumstances, like mate choice sexual selection, organisms did select some variants over others, and that these were especially important in current human evolution [1].

From an applied perspective, the distinction between agentic and non-agentic forces is fundamentally important. Waring *et al*. [2] introduced a multi-level selection framework for sustainability analysis. This terminology, to our taste, does not sufficiently emphasize the distinction between agentic and not-agentic forces. In general, policy-making is about using human choice, particularly collective choice, to change individual behaviour and institutions so as to avoid the effects of non-agentic natural selection. Cultural group selection in the form of violent conflict resulting in conquest and forced assimilation of conquered populations has been an important force in human history [3]. Although political elites often choose to make war, the outcome of violent conflicts has a strong element of non-agentic natural selection. In the aftermath of the bloody World Wars in the first half of the twentieth century policy makers innovated institutions such as the League of Nations and the United Nations (UN) to try to avoid wars and mitigate their harms, with some, but far from complete, success. Similarly with threats to sustainability, policy makers are using collective agency to devise international organizations to mitigate things like anthropogenic climate change, for example the UN Framework Convention on Climate Change, with some, but so far inadequate, success. Policy-makers want to deploy specific forms of *artificial* selection to try to avoid the worst effects of *natural* selection.

### (a) Non-agentic forces

Random processes affect genetic and cultural evolution. In the case of culture, memories are fallible and learners may not correctly understand their models. Cultural ‘mutation’ rates are fairly high in many domains, perhaps one of the reasons that cultural evolution is faster than genetic evolution [4]. In small populations, genetic or cultural variant frequencies can vary from time to time owing to random sampling errors. In some situations, some cultural models may have outsized influence, shrinking the effective size of the population. Even deleterious variants can increase in frequency by chance in small populations.

Any kind of heritable variation can be subject to natural selection. There is nothing special about genes in this regard. For example, Polynesian explorers undertook long, hazardous voyages to discover uninhabited islands in the Pacific to colonize. It is entirely plausible that voyagers using better boat designs or commanding better navigational techniques would more often survive these journeys, living to pass on the superior variants relative to those using inferior variants [5].

### (b) Agentic forces

In the case of cultural evolution, learning and creativity become forces of cultural evolution. The behavioural variants that one person discovers or invents can be passed on to others. Unlike genetic mutations these changes are not necessarily random with respect to fitness or other measures of function. Higher cultural than genetic mutation rates can be tolerated to the extent that they are better than random.

Social learners are also more or less discriminating adopters of variants they observe in their cultural models [6]. Social learners use many strategies for biasing their social learning to increase their chances of acquiring functional variants [7]. The simplest of these is trying out two or more variants and using the one with the highest pay-off. If pay-offs are hard to evaluate the social learner can use rules of thumb like adopting to most common local variant or adopting the recommendations of an expert.

Darwin [8] invoked tribal scale group selection in ‘primordial times’ as having favoured prosocial dispositions like sympathy and patriotism. Richards [9] argues that Darwin built a plausible theory of progressive reform based upon these ancient dispositions. There is, in other words, an element of prosociality in post-primordial agentic evolutionary forces. For example, cultural agents can also exert social selection on others by selecting individuals with one cultural (or genetic) variant rather than others as partners. In selecting a team for a cooperative venture, a team leader will usually select team-mates who are cooperative and skilled and expel those who prove troublesome or inept. A successful, skilled, and cooperative team will tend to produce higher pay-offs that in turn boost the cultural influence of the skilled and cooperative relative to the inept and troublesome. Similarly, voters in democratic systems seem often to favour prosocial candidates and propositions rather that self-interested ones [10].

Michael Tomasello [11] gives a good evolutionary account of the origins of human collective agency. The agentic forces are rooted in ancient decision-making abilities of organisms. Reinforcement-based learning is widespread [12]. Mate choice, partner choice, food choice and the like are similarly widespread. Choices can be collective. Humans use language to formalize rules and to persuade others of the desirability or undesirability of alternate rules, leading to our unusual capacity for collective decision-making to shape the evolution of institutions.

## 3. The evolution of the Anthropocene

Recently, our species has come to have major effects on the Earth's geochemistry and geophysics [13]. For example, industrial production has approximately doubled the Earth's fixed nitrogen income [14]. Crutzen argued that these impacts are leaving traces in the geological record that will be easily detectable far into the future. As such, the human dominated Epoch of geological history should be recognized with a geological term—the proposed the ‘Anthropocene.’ Human use of large-scale fire to manage vegetation in semi-arid areas goes back tens of thousands of years, as does the record of hunting impacts on the fauna [15]. On the other hand, industrialization, population increase, and fossil fuel exploitation in the last couple of centuries have dramatically increased our influence on Earth processes, leading Crutzen to propose this period as the beginning of the Anthropocene.

The Anthropocene is late in human evolution. Our genus *Homo* goes back about 2.5 million years, and our species *Homo sapiens* is about 300 000 years old [16]. For most of that time humans have been rather ordinary, even rare, organisms [17]. Why is the Anthropocene such a recent phenomenon? The answer, we believe, lies in the dynamics of cumulative cultural evolution (CCE) [18]. Under some circumstances, the processes of cultural evolution and gene-culture coevolution can lead to a situation where cultural sophistication can grow hyper-exponentially. The requirements for CCE seem to be, on the cognitive side, the ability to replicate culture accurately via high fidelity, selective imitation and teaching, and, on the population side, large interconnected social networks. If the fidelity of cultural transmission is too low, individuals have to spend too much time and effort reconstructing their phenotypes using individual learning, limiting the size and complexity of their cultural repertoires. If social networks are too small, social learners are exposed to only a few cultural variants and superior innovations cannot spread quickly. They are also are at risk of being lost by chance. CCE typically results in larger cultural repertoires and more complex artefacts and institutions, but the master criterion is greater utility not size and complexity *per se* [19,20]. General introductions to CCE include [21–25].

It is common to think of the history of humans as a gradual, relatively steady progress in our cognitive and cultural sophistication [26]. However, our actual evolutionary history seems to have been more complex. For example, around 50 000–70 000 years ago (50–70 kya), well after human physical form was very similar to ours and our cultures fairly complex, our numbers were still quite small [27,28]. Such anomalies suggest that we should pit the progressive account with ones that account for human evolution as a response to environmental changes.

### (a) The Pleistocene environment and the evolution of cumulative cultural evolution

There were dramatic changes in the Earth's environment during our evolutionary history [29]. The Cenozoic was characterized by a complex pattern of cooling, drying, and increasing variability of the environment caused by seafloor spreading, which rearranged the drifting continents to isolate the poles from warm ocean currents. The cooling of the polar regions generated a strong pole to equator temperature gradient that powered increasing climate variation. In the late Miocene, the African bipedal apes that became our ancestors evolved as deciduous forests, savannahs, and deserts expanded and the great apes' wet forest habitats contracted. This climate trend eventually culminated in the Pleistocene ice ages that featured unstable ice-covered polar regions and alternating glacial-interglacial climate fluctuations [30]. Beginning about 2.6 million years ago, the ice began to fluctuate mainly on the 41 thousand-year (41 ky) rhythm of the tilt of the Earth's axis. Geologists mark this as the beginning of the Pleistocene Epoch. Early members of our genus *Homo* evolved from the Pliocene bipedal Australopithecines around the beginning of the Pleistocene. A million years ago, the dominant glacial-interglacial pattern shifted to a 100 ky cycle tuned by the ellipticity of the Earth's orbit. Larger brained and culturally more sophisticated species of *Homo* evolved in this period, but there is no evidence that they became even common until the very end of the Pleistocene [31].

Superimposed on the relatively low frequency glacial-interglacial pattern were higher frequency millennial and submillennial scale variations, first resolved in the 1990s in Greenland ice cores going back to the last interglacial over 100 kya [32]. Subsequent high-resolution cores have pushed the record of the high frequency events back as much as 800 ky [33,34]. These data suggest that the high frequency variation has been increasing over time.

The millennial and submillennial scale variation is particularly interesting in the context of human evolution [35]. The time scales over which costly capacities for individual learning and social learning seem, from theoretical considerations, to stretch from generational and subgenerational to millennial time scales. Variation at the orbital variation scales (21, 41 and 100 ky) were probably met with genetic evolution and niche chasing via range changes. CCE in humans is faster than genetic evolution [4] and thus better able to track millennia and submillennial scale variation. The pattern of increase in cultural sophistication and brain size increase is similar to the pattern of increase in millennial scale variation over the last 400–800 ky. Thus, it is easy to imagine on theoretical and empirical grounds that the human capacity for cumulative culture evolved as a response to the increasing amount of high amplitude climate variation in the Pleistocene. This hypothesis will be further tested as the high-resolution climate and palaeo-ecological records are recovered back deeper into the Pleistocene and Pliocene [36].

Evolution in response to the externally imposed climate variation cannot be the whole story because, as important as culture, even cumulative culture, is in many species, the huge dependence of humans on culture is a distinct outlier [37]. The high metabolic cost of large brains and the need for an extended developmental period to make them useful slows the reproductive rate of large-brained species like our great ape cousins, whose maximum population growth rates are very low [38]. Humans evaded ape limitations on brain size by the expedient of cooperative breeding. Humans use kinship and friendship to mobilize males and pre- and post-reproductive females to provision mothers and their dependent offspring [39,40], substantially shortening our inter-birth intervals compared to other apes. As a consequence, our species has the potential for substantial rates of population growth if resources are plentiful.

The economic basis of the cooperative breeding system is technology fuelled subsistence systems that use tools and other technological practices such as cooking to exploit extracted and hunted resources, often using cooperation on a more or less large scale [41,42]. The great apes have two conspicuous preadaptations that put them on the edge of sustaining CCE. These include large brains and large social groups [43]. Large brains appear to be necessary to manage the cognitive demands of cumulative culture. Large social groups provide the personnel for cooperative activities and act to facilitate innovation, the conservation of cultural complexity and the rapid diffusion of advantageous innovations owing to biased horizontal and oblique cultural transmission [44,45]. Chimpanzee mothers dominate social transmission to their offspring, limiting chimpanzee's ability to use a large social network as a basis for selecting and diffusing superior cultural variants [46]. The evolution of specialized economic roles for males would have been difficult until males began to be social fathers and mentors to their younger male relatives and friends [47]. The evolution of cooperation in humans was arguably augmented by cumulative culture itself because cultural variation can support group level selection in a way that is difficult to imagine for genetic variation [48].

The Australopithecines added to this mixture free hands that were becoming specialized for the human power grip that is essential for such activities as pounding and knapping stone to make tools. The biomechanics of hands are well understood [49]. Stone cutting tools that can be made with human, but not other primate hands, are especially important because they are powerful machine tools that can be used to shape wood, cut fibrous material, and shape skins to make a large number of other tools [50]. Hands and the tools they make and use are fundamental to our exploitation of diverse culture-based socio-technological niches [51].

Thus, we have a two-part hypothesis to explain the origin of the human cumulative cultural adaptation. One part is the increasing millennial and submillennial scale climate variation that favours adaptation by learning and social learning. The other part is the ape and Australopithecine preadaptations of large societies and hands that could be specialized for toolmaking, uncompromised by the use of the forelimbs for locomotion. The large economic pay-offs to using tools to acquire difficult resources and deploying cooperation for subsistence practices meant that only humans could take massive advantage of the cultural niche [52]. A number of authors over the years have proposed that these preadaptations set up a positive feedback cycle that led to modern *H. sapiens* (e.g. [53]). However, the progressive increase in brain size and cultural sophistication in most of the Pleistocene does not look like a positive feedback process. The pace of progress seems more or less linear and to have been paced by the ongoing increase in high frequency climate variation [35]. The increasing overhead costs of large brains seems to have damped any positive feedback runaway process for a couple of million years. As anatomically modern humans expanded their range out of Africa perhaps 70 kya, mitochondrial DNA coalescence time data suggest populations were still very small, although numbers would shortly begin to increase [28]. The cognitive and social preadaptations necessary for CCE seem to have existed for tens of millennia before the Anthropocene runaway began in earnest.

### (b) Latest Pleistocene: hinge of the Anthropocene

Anatomically modern human populations seem to have begun a steady increase about 50 kya, with rather different patterns in different continents and subcontinents of Eurasia [27,54]. This change in human demography is coincident with *H. sapiens* expansion out of Africa to fairly shortly becoming the only living human species. Possibly, a fortuitous mutation in a gene influencing human creativity sparked a sustained increase in the rate of cultural innovation [55]. A major problem with this explanation is that our sister species, the Neanderthals, also seem to display signs of a cultural acceleration late in their evolutionary history more or less coincident with the acceleration in *H. sapiens* [56]. There is also a plausible environmental explanation for the increased rate of cultural cumulation beginning around 50 kya. The last half of the last ice age had the highest rate of millennial scale variation so far observed in the palaeoclimate record [33,34]. Such an increase in high frequency climate variation plausibly gave highly cultural large brained humans a decisive advantage over their minimally cultural top carnivore competitors. The resulting increase in human population would increase rates of cultural innovation [57,58]. Humans finally seem to have become dominant carnivores leading, among other things, to a major megafaunal extinction event [59], first in Eurasia and Australia, then in the New World after humans reached it perhaps 15 kya.

### (c) The Holocene and the evolution of agriculture

The high frequency climate variation characteristic of ice ages ameliorated rather abruptly after 11 700 years ago. The Earth also became, on average, warmer and wetter, and the concentration of CO_2_ in the atmosphere increased. These factors were conducive to the development of agriculture and, more generally, to plant intensive subsistence systems [60]. Agriculture is a highly significant innovation because it replaces the predator–prey relationship to the species humans subsist upon with a mutualistic one. Cumulative culture tends to create human super-predators that collapse exploited species, as the end Pleistocene megafaunal extinctions illustrate, whereas CCE tends to fuel the explosive growth of farming subsistence ecosystems [61,62]. It is sometimes said that the origin of agriculture produced an immediate demographic transition, but recent quantitative evidence suggests a different picture. The population growth rates of early Fertile Crescent farmers were very similar to the growth rates of western North American hunter–gatherers fairly deep into the Holocene [63]. In the Holocene, both farmers and hunter–gatherers were slowly evolving more plant rich subsistence systems, and the explosive potential of the farming mutualism is hard to detect empirically until the middle Holocene. The curve of global human population increase has a pronounced hockey stick pattern with a long tail of quite slow, but slowly accelerating, growth that conspicuously explodes in an accelerating fashion approaching the present [64]. In the last couple of centuries, the modern demographic transition has progressively reduced population after population to replacement, even below replacement, fertility, but rising consumption per capita has meant that human pressure on other species and on ecosystem services has continued to increase at a rapid rate.

The hockey stick pattern can be approximated by fairly simple coupled differential equation models that introduce multiple positive feedbacks [64]. In Genet *et al*.'s [62] model, a growing human population increases the rate of cultural cumulation on the argument that larger populations will have more innovators [44]. Culture also cumulates exponentially in their models on the argument that many innovations are recombinations of existing innovations, so the larger the stock of techniques and designs, the easier innovation becomes. The more complex models introduced by Efferson *et al*. [65] exhibit a wide range of behaviour depending on parameter values and initial conditions. Notably, the commonest stable state is a ‘post human’ equilibrium in which humans and their culture disappear. The ‘natural’ models of humans with cumulative culture constructed so far, at least, are inherently unstable. Sustainable human systems would seem to require large-scale adaptive management to be sustainable.

The coupled differential equation models are deterministic but they do incorporate an agentic innovation process in the sense that humans are motivated to innovate by resource shortfalls and to adopt the innovations of others. They are blind to the large-scale problems generated by runaway CCE and lack the element of group-level strategic decision-making that is likely where solutions to the problems of the Anthropocene are to be found. They are models of business-as-usual in the jargon of the International Panel on Climate Change reports.

## 4. Application of cultural evolution to the problem of sustainability: limitations and prospects

The concepts and methods of evolution constitute one of the most important synthetic principles in the science of living things [25]. As such, it may seem as if evolution should play a central role in the applied science of sustainability. In the sense of basic science, this ambitious claim is, we think, warranted. We submit our foregoing CCE explanation for the Anthropocene as evidence for this fundamental role. However, as an applied science it has limitations as well as promise. We believe that applied cultural evolutionists need to think strategically about exactly where they can make useful applied contributions, where they either have no useful advice, or where other disciplines have a comparative advantage.

A useful analogy is evolutionary medicine [66], where advocates have had trouble convincing the medical establishment of its utility [67,68]. In evolutionists' terms, many questions of practical medical treatment involve proximal causes, while evolution deals mainly with ultimate causes. Proximal causes involve the way physiological, anatomical, and behavioural mechanisms accomplish ultimate functions related to survival and reproduction [69]. Typically, proximal mechanisms are only loosely constrained by ultimate imperatives. An extreme example is symbolic behaviours like language or sexually selected displays. Language has vital communication functions without which human life as we know it is not possible. Yet, to a first approximation, all 7000 of the world's languages serve these essential functions equally well using highly diverse vocabularies and syntaxes. Even in much more constrained domains the variation in proximate factors is considerable. For example, drug development is heavily dependent on model organisms, especially the mouse. However, candidate drugs that work well in mice often fail in human clinical trials. Mice are very convenient (cheap) models of humans, they are fairly closely related to us. However, the genetics and physiology of mice are appreciably different from that of humans so they are far from perfect models [70]. Humans are not genetically very variable, yet the database on clinically relevant human variation is quite large [71]. Human cultural variation is at least an order of magnitude larger than our genetic variation [72], much of that is most likely not a result of identifiable ultimate factors [73].

Most of the applied social sciences, like most of medical science, operates on the basis of what we think of as a naive functionalist model. Society, like the body, can be analogized to a machine that not infrequently malfunctions. Applied science consists of identifying the usually proximal cause of malfunction and repairing it. Applied history, economics, political science, sociology, and anthropology mostly follow the naive functionalist approach to problems, and, in their willingness to deal with proximate causes of problems, they have a comparative advantage over evolutionists. For example, if you want to convince a skeptical audience of the use of a proposed solution to a problem, you are better off consulting the ancient art/science of rhetoric (persuasion) than evolutionary theory. Where evolutionists do have a comparative advantage is in issues where population dynamics are important. The recent Covid-19 pandemic highlighted, on the one hand, how proximal biology, as in the rapid development of vaccines, had a vital role to play. On the other hand, epidemiology (models of ecological and evolutionary dynamics of disease) was useful to predict the course of the pandemic and the evolution of the virus. For example, the evolution of the relative avirulence of successive strains of SARS-CoV-2 is well predicted by epidemiological theory ([74], P. W. Ewald 2023, personal communication). The virus should evolve to be about as lethal as the seasonal flu, as successive strains actually have.

We encourage human ecologists and cultural evolutionists interested in applications to sustainability and similar real-world problems to think of their subject as the cultural branch of epidemiology. The distinctive contribution of applied cultural evolution is also likely to mostly lie in the dynamic tools that evolutionists can bring to problems. On the theoretical side, recursion equations and coupled differential equations are conceptually and mathematically closely related to epidemiological models. The idea that elements of culture (memes) can spread like biological pathogens was introduced by Dawkins [75]. Richerson & Boyd [76] used evolutionary game theory to model evolution in a situation where cultural variation could favour or disfavour genetic fitness to varying degrees. Several cultural-evolutionary processes can lead to pathological cultural elements evolving. For example, group selection on culture can lead to large scale cooperation in human societies with high pay-offs to participating individuals. At the same time, selection on genes tends to operate on smaller scales, giving rise to selfish motivations. Much of the political drama in human societies pits public virtue against private vice. Boyd & Richerson [77] showed how prestige biased cultural transmission might give rise to pathological runaway cultural evolution. These models might be applicable to understanding the growth of the QAnon political conspiracy theory and similar ideological extremisms, some of which vex efforts to address sustainability issues. The subdivision of humans into ethnicities, classes, castes, religions, economic organizations, and the like can give rise to conflicts, sometimes quite debilitating ones. Peter Turchin [78] has used coupled differential equation models of class-stratified societies to understand the rise and fall of political discord. Efferson *et al*. [65] introduce a model that blends elements of ecology, economics and cultural evolution. These models roughly fit the empirical data. They are perhaps alarming in that they have no stable equilibrium with humans present in the system. Turchin's and Efferson's models are deterministic, lack policy level feedback control, and have alarming future trajectories. To the extent that such small-minded robotic models fit the data, they suggest that policy-makers need to innovate control strategies to counter the instability inherent in CCE.

In principle, any institutional innovation designed to promote sustainability can generate both individual and collective forms of behaviour change. Some people will fall under the immediate sway of the new institution and the incentives it creates, and some of these people may change behaviour as a result. This kind of behaviour change constitutes the direct effect of the intervention. Once people start to change behaviour in this way, indirect effects may also occur as people learn new ways of thinking and behaving from each other, and endogenous cultural evolution takes over. Depending on the details, for example, conformity and coordination incentives may lead to socially beneficial changes in behaviour that are consistent with, but far outstrip the original ambitions of the institutional innovation. The smart social planner designs institutions that maximize direct effects, and she implements institutional change in a way that also maximizes indirect effects. The idea that the well-intentioned social planner can recruit cultural evolutionary processes to promote beneficial changes in behaviour has generated enormous interest as a way to support conservation and mitigate climate change [79–83]. A key challenge, however, is that ordinary forms of heterogeneity can dramatically affect how well this works [84,85]. Imagine, for example, that everyone is a conformist, but they also vary in terms of their preferences. This mundane scenario creates a fundamental but poorly understood trade-off. An institutional innovation that focuses on those amenable to sustainable choices would maximize the direct effect but minimize the indirect effect. If instead, the innovation focuses on those resistant to sustainable choices, it will minimize the direct effect but maximize the indirect effect conditional on the direct effect that does occur [86]. Maximizing the total effect requires the social planner to manage the trade-off these two countervailing forces induce.

On the empirical side, evolutionists bring useful tools to study short term and long-term dynamic processes. Laboratory microcosms can be used for short term cultural evolution [87], including asking ambitious questions [88]. In especially favourable circumstances a rather detailed understanding of real-world evolution is possible [89]. With sufficient resources, sophisticated field experiments can be used to test the efficacy of cultural-evolution-based interventions [90]. Evolutionary game theory has been applied to the study of cross-cultural variation in cooperative performance of different societies [91,92], suggesting that it might be used to diagnose the institutional strength of organizations in applied contexts. The development of historical databases promises to greatly improve causal analysis of medium and long-term cultural evolutionary phenomena [93]. Similarly, large scale multi-wave survey research provides data on cultural changes. From an applied point of view, rates of cultural evolution are obviously critical to policy debates. If favourable cultural trends are rapid relative to the magnitude of a problem, we might be justified in avoiding costly or risky policy changes. For example, China's draconian one-child policy was unnecessary because the Chinese demographic transition was already rather advanced when it was instituted. More generally the demographic transition has substantially defused the population bomb that worried previous generations of activists [94]. On the other hand, if they are slow and unfavourable relative to the magnitude of the problem, measures to increase the rate and direction of cultural change may need to be taken. For example, climate sensitive strategic public and private investments in wind, solar, and battery technology have been dramatically successful, to the point that decarbonizing the energy system now seems possible. However, the mass demand for affluent consumption patterns on the part of the long-time affluent and on the part of some large, rapidly growing, formerly poor societies suggests that heavy pressure on planetary limits will continue even if rapid decarbonization does succeed. The drive behind pro-affluence policies based on simply increasing gross domestic product/capita is arguably pathological on its own terms, the burden it places on environmental resources aside [95].

A full working out of the cultural epidemiology idea is beyond the scope of this essay, but two foundational points are worth mentioning. First, narrowing the primary ambition of applied cultural evolution to our most distinctive set of tools avoids giving the impression of disciplinary imperialism that is off-putting to those in our sister disciplines that may have a better grasp of answers to many applied questions than we do. Inevitably, applied (and basic!) scientific problems demand an expedient and eclectic mix of expertise across a range of fields. We have a contribution to make but so do ‘they.’ Second, we have spoken glibly of good and bad cultural variation here. Of course, one person's cultural pathology is often another's true belief. Applied science should have a self-conscious ethical dimension [96]. Applied science usually results in a case for changing human behaviour or retaining current practice in the face of proposals for change. Some applied science advice, like recommending the aforementioned vaccine in the face of the pandemic, seems ethically rather simple but proved controversial in some quarters. Applied science often requires ethical sensitivity and, sometimes, moral courage.

## 5. Institutional evolution by agentic processes

An extremely important form of CCE from the point of view of achieving sustainable economic systems is the agentic evolution of institutions. Institutions are generally defined as culturally transmitted systems of rules that organize human social life [97]. Since even the simplest known human societies are governed by institutions like kinship and marriage, the evolutionary roots of institutions are probably 50 ky old, if not far older [98]. Institutions evolve by both agentic and non-agentic processes. Again, even simple societies have deliberative political institutions that lead to the evolution of rather sophisticated collective behaviour [99,100]. However, non-agentic processes also play an important role, even in complex societies. Institutions play a strong role in determining the outcome of intergroup competition and so group level selection plays a strong role in, for example, the success and failure of competing modern corporations and churches [48]. The dominant pattern of institutional evolution in the Holocene has been the evolution of ever more complex institutional systems, culminating in the modern bureaucratic states organizing many millions of people [101] and agentic forces have played a very large role in these trends. We have even engineered non-trivial global institutions and organizations based on these institutions, for example the UN Framework Convention on Climate Change.

The global scale commons problems presented by anthropogenic climate change, the biodiversity crisis, weapons of mass destruction, inequality and the like would seem to require us to mobilize collective agency to create institutional solutions to solve the large-scale dilemmas of cooperation they present. In a science-fiction world, interplanetary competition might solve such problems by group selection, if at a terrible cost to the competitive losers. As it is, we are presumably on our own. Individual agency is of real value, even in the face of global problems. We can change our own behaviour but the dilemma of cooperation, exemplified on the laboratory scale by the Prisoners Dilemma and Public Goods Games, requires *collective* agency. The dynamics of cultural change by collective agency are not as well studied by cultural evolutionists as change owing to individual agency. We inherit suboptimal institutions, such as the powerful, competitive, modern nation state, from a time when global scale commons problems were less extreme. Twentieth century failures to solve international security issues in the face of industrialized warfare and the development of weapons of mass destruction stimulated hard thinking and non-negligible innovative action on that collective problem. The problem of environmental sustainability doubles down on the need for effective large-scale collective action if we are to evade the costs of failing to solve the climate change and biodiversity problems.

The mechanisms by which institutions evolve by collective action has received much scholarly attention [102–104], mostly by economic historians and political scientists. A toy power-weighted-majority-vote model of political process by which institutional change happens summarizes much of this literature. Imagine a policy arena which is governed by an existing institution or set of institutions. Entrepreneurial individuals or small groups propose changes to the existing rules for some mixture of self-interested and public interested motivations. They attempt to recruit a larger party to support their proposed innovation using advocacy and persuasion. Conservatives defend the existing arrangement for a similar mix of motives. Usually the costs and benefits of existing arrangements are better known than those of the proposed innovation. Human and physical capital have been largely shaped by existing institutions and change will generate transition costs. In spite of this inertia, many people may come to favour the innovation, often because some process like cumulative technological evolution is changing the cost-benefit calculation fairly decisively in favour of the innovation. If all were equal participants in the political process, a simple majority vote would decide the adoption or not of the innovation. In practice, the power and influence of the actors in the political system is never perfectly egalitarian and, in many systems, the formal franchise may be quite narrow, down to a single individual in the case of tyrannies. This model helps explain why institutional innovation is often punctuational with much change concentrated in the short spans of time. Even if underlying individual attitudes are changing gradually and smoothly, institutions will not change until innovators reach the threshold of commanding the balance of political power. Gay marriage rather suddenly became possible when a majority of voters and the elite alike came to favour it. Polling suggests that a majority of citizens support action on climate change [105] but the political power of the fossil fuel industry and its supporters remains sufficient to block more than incremental action. Nevertheless, a fierce attempt to attack its legitimacy and power is ongoing and making headway [106]. Paul Sabatier's Advocacy Coalition/Policy Learning framework is an explicitly evolutionary approach elaborating the toy model, and Ostrom's Institutional Analysis and Development framework is more naive-functionalist, but both lead to practical guidance for action [107] that should be as applicable to sustainability as to other policy domains.

## 6. Conclusion

The cultural evolutionary/gene-culture coevolution framework provides a reasonably clear and complete set of hypotheses to explain the advance of the Anthropocene as a process. Most of these hypotheses are contested and not all uncertainties are resolved.

The process cumulative culture generated the technologies and social organizations that caused humans to become a geological force unfolded in a nonlinear but more or less monotonic way from about 50 kya onwards. A formal geological definition of the beginning of the Anthropocene could be based at the earliest on the onset of the megafaunal extinctions around 40 kya or by the appearance of diverse and easily detectable industrial waste products in sedimentary deposits around 50 ya at the latest. There is an inevitable arbitrariness in periodizing a continuous process.

The toy model of institutional evolution, and the large body of work on institutional evolution by political scientists, historians, and other social scientists that it is based upon, should help shape a strategy for cultural evolutionists to contribute to the applied science of sustainability and other areas of policy making. It seems to us that the comparative advantage of the evolutionary sciences lies in their conception of human societies as culturally evolving systems operating in a dynamic environment. Evolutionary causes are iteratively extended in time, leading to lags, overshoots, coevolutionary responses, sensitivity to initial conditions and similar complex temporal patterns that evolutionists are better equipped to understand than other scientists. A simple lesson based on these considerations is that applied scientists working in the policy arena to be successful must engage for the long haul. Attempts by scientists to have impacts on the policy process with one-shot injections of expert opinion are hardly ever successful [107].

## Data Availability

This article has no additional data.
